# Increasing outer membrane complexity: the case of the lipopolysaccharide lipid A from marine *Cellulophaga pacifica*

**DOI:** 10.1007/s10719-024-10149-8

**Published:** 2024-04-20

**Authors:** Emanuela Andretta, Stefania De Chiara, Chiara Pagliuca, Roberta Cirella, Elena Scaglione, Martina Di Rosario, Maxim S. Kokoulin, Olga I. Nedashkovskaya, Alba Silipo, Paola Salvatore, Antonio Molinaro, Flaviana Di Lorenzo

**Affiliations:** 1 Department of Chemical Sciences, University of Naples Federico II, via Cinthia, 4, Naples, 80126 Italy; 2 Department of Molecular Medicine and Medical Biotechnologies, University of Naples Federico II, Via S. Pansini, 5, Naples, 80131 Italy; 3 Far Eastern Branch, G.B. Elyakov Pacific Institute of Bioorganic Chemistry, Russian Academy of Sciences, 159/2, Prospect 100 Let Vladivostoku, Vladivostok, 690022 Russia; 4 CEINGE-Biotecnologie Avanzate Franco Salvatore, Via G. Salvatore, 436, Naples, 80131 Italy; 5 Task Force on Microbiome Studies University of Naples Federico II, Naples, 80100 Italy

**Keywords:** Marine bacteria, *Cellulophaga*, Lipopolysaccharide, Lipid A, MALDI-TOF MS, Innate immunity

## Abstract

Gram-negative bacteria living in marine waters have evolved peculiar adaptation strategies to deal with the numerous stress conditions that characterize aquatic environments. Among the multiple mechanisms for efficient adaptation, these bacteria typically exhibit chemical modifications in the structure of the lipopolysaccharide (LPS), which is a fundamental component of their outer membrane. In particular, the glycolipid anchor to the membrane of marine bacteria LPSs, i.e. the lipid A, frequently shows unusual chemical structures, which are reflected in equally singular immunological properties with potential applications as immune adjuvants or anti-sepsis drugs. In this work, we determined the chemical structure of the lipid A from *Cellulophaga pacifica* KMM 3664^T^ isolated from the Sea of Japan. This bacterium showed to produce a heterogeneous mixture of lipid A molecules that mainly display five acyl chains and carry a single phosphate and a D-mannose disaccharide on the glucosamine backbone. Furthermore, we proved that *C. pacifica* KMM 3664^T^ LPS acts as a weaker activator of Toll-like receptor 4 (TLR4) compared to the prototypical enterobacterial *Salmonella typhimurium* LPS. Our results are relevant to the future development of novel vaccine adjuvants and immunomodulators inspired by marine LPS chemistry.

## Introduction

Marine environments host a wide variety of microorganisms that represent a rich source of natural substances able to exert immunomodulatory, anti-tumor, antibacterial and/or antimicrobial properties [1–8]. Most of these molecules are produced by microbes in response to the extreme conditions of temperature, salinity and/or pressure, typical of marine waters, which have boosted weird forms of adaptation to ensure microbial survival [9, 10]. An apt example is given by Gram-negative bacteria that have developed peculiar structural modifications in their cell envelope and its components, particularly in the lipopolysaccharide (LPS) moiety, to cope with the stressors of deep sea and coastal marine habitats [11–15]. The surface of Gram-negative bacteria is in fact made up of an asymmetric membrane that contains LPS on its external leaflet, which acts as a potent barrier impermeable to many dangerous compounds, thus providing high resistance to a wide range of external stressors. An LPS molecule typically consists of three components: a glycolipid anchor to the outer membrane (the lipid A), an oligosaccharide portion (termed core oligosaccharide), and a polysaccharide chain (the O-antigen) [14, 16]. The presence of all these three moieties defines an S-LPS (or smooth-type LPS), while the absence of the O-antigen is typical of an R-LPS (or rough-type LPS) [14, 17]. All these structural moieties can be affected by modifications dictated by environmental pressures. In particular, structural alterations can occur in the lipid A region and usually include desaturation of the acyl chains, increase in their branching, and reduction in their length, as well as modification in the phosphorylation degree [15, 18–22].

From an immunological point of view, LPS is traditionally acknowledged for its capability to stimulate potent inflammatory reactions in mammals, mediated by its crosstalk with the innate arm of the immune system [14]. The receptorial complex made up of Toll-like receptor 4 (TLR4) and myeloid differentiation factor-2 (MD-2) is the key interlocutor of LPS as it specifically recognizes its lipid A moiety [23]. The activation of TLR4/MD-2 complex results in the induction of NF-kB signaling and the release of chemokines and inflammatory cytokines [14]. As a matter of fact, the immune system overstimulation induced by LPS of pathogenic bacteria has been associated with life-threatening events in humans, such as sepsis and lethal sepsis shock [14, 24, 25]. However, it is worth underlining that the chemical structure of lipid A strongly influences the degree of this dangerous TLR4/MD-2-dependent inflammatory response. Therefore, LPSs displaying different chemical features in their lipid A moieties can exhibit largely diverse immune inflammatory reactions and thus can behave as weak or strong TLR4 agonists or even as TLR4 antagonists [24]. In this regard, several studies have pointed out that a huge number of LPSs and lipid As with uncommon structural features, such as those observed in marine bacteria, display weak immunostimulatory properties and, in some cases, even an inhibitory effect (i.e. an antagonistic action) against potent inflammatory LPSs [22, 26, 27]. Suffice is to say that LPSs from marine bacteria are considered promising candidates for treatment of economically important immune-mediated diseases [20].

In this perspective, we have previously reported about the lipid A structure and weak TLR4-agonistic activity of LPS from three marine bacteria of *Cellulophaga* genus, i.e. *C. algicola* ACAM 630^T^, *C. tyrosinoxydans* EM41^T^ and *C. baltica* NNO 15840^T^ [28]. In this study we focus on *C. pacifica* KMM 3664^T^ that was isolated from a sea water sample collected in Amursky Bay, Gulf of Peter the Great, Sea of Japan, Pacific Ocean, from a depth of 5 m [29]. We report the structural characterization of the lipid A moiety obtained by merging data from chemical analyses and the use of Matrix-Assisted Laser Desorption/Ionization-Time of Flight Mass Spectrometry (MALDI-TOF MS) and tandem MS (MS/MS). We show that *C. pacifica* KMM 3664^T^ produces a heterogeneous family of tetra- to hexa-acylated lipid A species mostly carrying one phosphate at position 1 of the reducing glucosamine and a disaccharide of D-mannose at position 4’ of the nonreducing glucosamine. To evaluate the immunostimulatory effect of this complex blend of lipid A species, *C. pacifica* KMM 3664^T^ LPS was also tested in HEK Blue hTLR4 cells stably transfected with human TLR4 (hTLR4) and MD-2/CD14 genes. Notably, the treatment with *C. pacifica* KMM 3664^T^ LPS induced a significantly weaker TLR4 activation compared to the pro-inflammatory *Salmonella typhimurium* LPS.

## Materials and methods

### Bacteria isolation and growth

*Cellulophaga pacifica* strain KMM 3664^T^ (= JCM 11,735^T^ = LMG21938^T^) comes from the Collection of Marine Microorganisms of the Pacific Institute of Bioorganic Chemistry, Far East Branch of Russian Academy of Sciences and was isolated from a sea water sample collected in Amursky Bay, Gulf of Peter the Great, Sea of Japan, Pacific Ocean, from a depth of 5 m [29]. It was grown for 48 h at ambient temperature on a medium consisting of (L^− 1^) 5 g Bacto Peptone (Difco), 2 g BactoYeast Extract (Difco), 1 g glucose, 0.02 g KH_2_PO_4_ and 0.05 g MgSO_4_‧7H_2_O in 50% (v/v) natural seawater and 50% (v/v) distilled water. Bacterial cells were then dried by acetone and prepared for LPS extraction.

### Extraction and purification of the LPS from *C. pacifica* KMM 3664^T^

Dried bacterial cells were extracted using the hot phenol/water procedure [30]. Traces of phenol and other contaminants were removed by an extensive dialysis against distilled water (Spectra/Por®, Fisher Sci. Leicestershire, UK, cut-off 12–14 kDa). Sodium Dodecyl Sulphate-PolyAcrylamide Gel Electrophoresis (SDS-PAGE), followed by silver nitrate-gel staining was performed to assess the nature and purity of the extracted LPS. Then, to remove cell contaminants, such as nucleic acids and proteins, an enzymatic treatment was performed using DNase (Sigma-Aldrich, Darmstadt, Germany), RNase (Sigma-Aldrich, Darmstadt, Germany) and protease (Sigma-Aldrich, Darmstadt, Germany), followed by dialysis against distilled water (Spectra/Por®, Fisher Sci., Leicestershire, UK, cut-off 12–14 kDa). Ultracentrifugation (200,000 × *g*, 4 °C, 16 h) and size exclusion chromatography on a high-resolution Sephacryl S-500 column (GE Healthcare, Buckinghamshire, UK) were also executed. To exclude the presence of phospholipids possibly contaminating the LPS extract, several washes with a mixture of CHCl_3_/CH_3_OH (1:2, v/v) and CHCl_3_/CH_3_OH/H_2_O (3:2:0.25, v/v) were accomplished on an aliquot of LPS. Following the removal of organic solvents, the LPS fraction was lyophilized and underwent a complete set of chemical analyses, which included the analysis via Gas Chromatography Mass Spectrometry (GC-MS) of fatty acids as methyl ester and methoxy acid L-phenylethylamide derivatives, as well as by GC-MS inspection of monosaccharides composing the lipid A backbone once converted into their acetylated methyl and acetylated *O*-(-)-2-octyl glycoside derivatives, as described below in more detail.

### Compositional analyses

Total fatty acid content was determined by treating an aliquot of LPS with 4 M HCl (100 °C, 4 h), followed by a treatment with 5 M NaOH (100 °C, 30 min). Fatty acids were then extracted in chloroform, after the adjustment of the pH (pH⁓3), methylated with diazomethane, and analyzed via GC-MS. Ester-bound fatty acids were released after treatment with aqueous 0.5 M NaOH in CH_3_OH (1:1, v/v, 85 °C, 2 h), followed by acidification of the products, extraction in chloroform and methylation with diazomethane. The obtained fatty acids were then inspected via GC-MS. In addition, another aliquot of LPS underwent methanolysis with 1.25 M HCl/CH_3_OH (85 °C, 16 h), then the mixture was extracted with hexane three times. The hexane layer, containing the fatty acids as methyl esters, was then analyzed via GC-MS [31]. Absolute configuration of the 3-hydroxy and 2-hydroxy fatty acids was assigned after their release through a treatment with 4 M NaOH (100 °C, 4 h) and conversion into methoxy acid L-phenylethylamide derivatives [32]. These were then analyzed via GC–MS. The retention times of authentic L-phenylethylamides of standard fatty acids were compared with those derived from *C. pacifica* KMM 3664^T^ lipid A. The analyses were all executed on an Agilent Technologies gas chromatograph 6850 A equipped with a mass selective detector 5973 N and a Zebron ZB-5 capillary column (Phenomenex, Torrance, CA, USA, 30 m × 0.25 mm internal diameter, flow rate 1 mL/min, He as carrier gas).

### Fractionation and analysis of the *C. pacifica* KMM 3664^T^ lipid A

An aliquot of purified LPS (20 mg) was treated with acetate buffer (pH 4.4, 3 h, 100 °C) under constant magnetic stirring. A mixture of CH_3_OH and CHCl_3_ was then added to the acid hydrolysis product to reach a CH_3_OH/CHCl_3_/hydrolysate 2:2:1.8 (v/v/v) ratio. This mixture was then shaken and centrifuged (4 °C, 8000 × *g*, 20 min). Then, the chloroform phase was collected and transferred in a clean tube where it was washed with methanol and water to reach a CHCl_3_/CH_3_OH/H_2_O, 2:2:1.8 ratio. This step was executed three times. All the organic phases were pooled, dried, and analyzed by MALDI-TOF MS. In addition, an aliquot of the isolated lipid A fraction was treated with 1.25 M HCl/CH_3_OH (80 °C, 16 h), followed by acetylation (80 °C, 20 min) and GC-MS analysis. Finally, the D-configuration of the mannose and the glucosamine composing the lipid A saccharide backbone was defined by GC-MS analysis of the acetylated *O*-(-)-2-octyl glycoside derivatives and comparison with authentic standards [33].

### MALDI-TOF MS and MS/MS analysis of *C. pacifica* KMM 3664^T^ lipid A

MALDI-TOF MS and the MS/MS experiments were all performed in reflectron mode, both positive and negative-ion polarity, on an ABSCIEX TOF/TOF™ 5800 Applied Biosystems (Foster City, CA, USA) mass spectrometer equipped with an Nd: YAG laser (λ = 349 nm), with a 3 ns pulse width and a repetition rate of up to 1000 Hz, and also equipped with delayed extraction technology. Lipid A was dissolved in CHCl_3_/CH_3_OH (50:50, v/v). The matrix used was 2,4,6-trihydroxyacetophenone (THAP) dissolved in CH_3_OH/0.1% trifluoroacetic acid/CH_3_CN (7:2:1, v/v/v) at a concentration of 75 mg/mL. 0.5 µL of the sample and 0.5 µL of the matrix solution were spotted onto a stainless-steel plate and left to dry at room temperature. For MS experiments each spectrum was derived by the accumulation of 2000 laser shots, whereas 5000–7000 shots were summed for the MS/MS spectra. All experiments were executed in technical triplicates.

### HEK cell culture

HEK-Blue™ hTLR4, HEK-Blue™ hTLR2, and HEK-Blue™ Null2™ cells (InvivoGen, San Diego, CA, USA) were cultured at 37 °C in 5% CO_2_ using Dulbecco’s minimal essential media (DMEM) supplemented with 10% heat-inactivated fetal bovine serum (FBS) (Microgem, Naples, Italy), 1% glutamine (Himedia Einhausen, Germany), 1% penicillin/streptomycin (Himedia, Germany), and 100 µg/mL Normocin (InvivoGen). Plasmid selection in HEK-Blue™ hTLR4 and hTLR2 cells required the use of a mixture of selective antibiotics (HEK-Blue™ Selection) (InvivoGen), whereas HEK-Blue Null2™ cells required the use of 100 µg/mL Zeocin (InvivoGen).

### HEK-Blue™ Null2, hTLR2 and hTLR4 stimulation assays

HEK-Blue™ hTLR4 cells were seeded into 96-well plates (3 × 10^5^ cells per well) and incubated with different concentrations of *C*. *pacifica* KMM 3664^T^ or *Salmonella typhimurium* SH 2201 LPS (1, 10, 100 ng/mL) for 18 h to analyze NF-κB activation by evaluating NF-κB-dependent secreted alkaline phosphatase (SEAP) using QUANTI-Blue™ (InvivoGen). Likewise, HEK-Blue™ hTLR2 and HEK-Blue™ Null2™ cell lines were treated with *C*. *pacifica* KMM 3664^T^ or *S. typhimurium* SH 2201 LPS (1, 10, 100 ng/mL) for 18 h and analyzed as above. HEK-Blue™ hTLR2 cells were also stimulated with Pam3CSK4 (500 ng/mL) (InvivoGen) that was used as the positive control for this assay.

## Results

### Isolation of LPS from *C. pacifica* KMM 3664^T^ and chemical analyses

LPS was isolated from dried cells of *C*. *pacifica* KMM 3664^T^ through the hot phenol/water procedure [30] that was followed by extensive purification by means of enzymatic digestion, dialysis, ultracentrifugation, and size-exclusion chromatography. An SDS-PAGE followed by silver-nitrate gel staining [34] was performed to evaluate the nature and degree of purity of the extracted material. The gel showed the typical ladder-like pattern of an O-antigen-expressing LPS (i.e., an S-LPS), which agreed with our previous analysis [29]. Fatty acid compositional analysis was executed on an aliquot of LPS (results are reported in Table 1), which evidenced the same astounding heterogeneity observed for other *Cellulophaga* strains and that entailed both 2- and 3-hydroxylated, branched, unbranched, saturated and unsaturated fatty acid chains [28].

**Table 1:** Fatty acid content of the lipid A isolated from *C. pacifica* KMM 3664^T^. All 3-hydroxy fatty acids displayed an (*R*) configuration while 2-hydroxylated 15:0 showed an (*S*) configuration. For the unsaturated acyl chains the position of the double bond or the stereochemistry remain to be established. Likewise, for *anteiso*-branched fatty acid chains the stereochemistry is tentatively given as (*S*) given its predominancy in bacteria; however, it remains to be defined

Fatty acid content	
12:0	16:0
*i*13:0	14:0 (3-OH)
13:0	15:0(2-OH)
*i*14:0	*i*15:0(3-OH)
14:0	*a*15:0(3-OH)
*i*15:1	15:0(3-OH)
*a*15:1	16:0(3-OH)
15:1	*a*17:1
*i*15:0	*i*17:1
*a*15:0	17:1
15:0	17:0
*i*16:1	*i*17:0(3-OH)
16:1	*a*17:0(3-OH)
*i*16:0	17:0(3-OH)

**Fig. 1:**
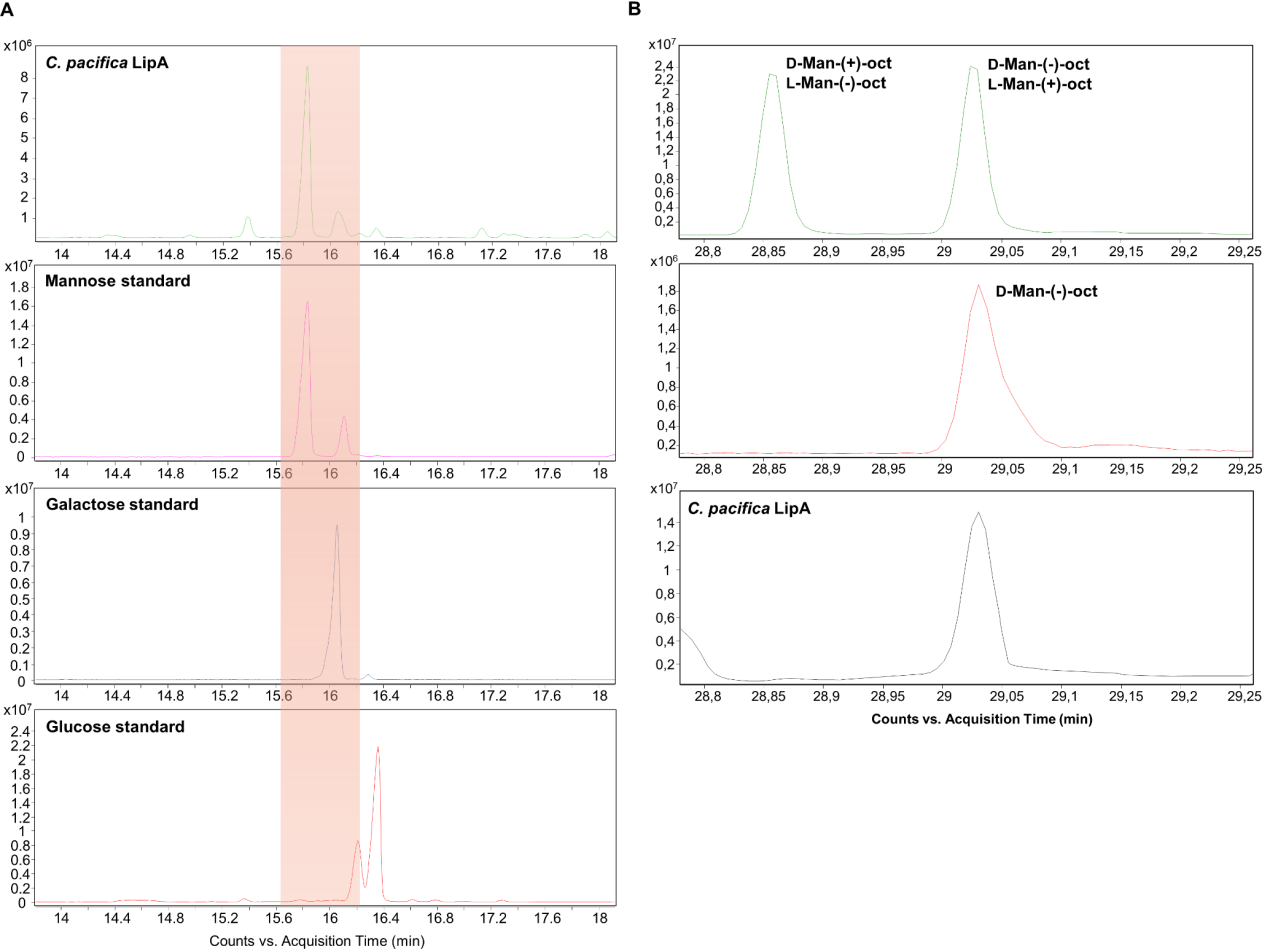
(**a**) Section of the GC-MS chromatogram profiles of the acetylated methyl glycoside of the hexose found in *C. pacifica* KMM 3664T lipid A and of mannose, galactose and glucose opportunely prepared from authentical standards and used as references. The comparison of the retention times clearly demonstrated that the unknown hexoses decorating the lipid A of *C*. *pacifica* KMM 3664T were all mannoses. (**b**) Zoom of the GC-MS chromatogram of the acetylated octyl glycoside derivatives of the mannoses found in *C. pacifica* lipid A which were generated by using 2-(-)-octanol. Parallel reactions of 2-(±)-octanol and 2-(-)-octanol with a standard of D-mannose (D-Man) produced a mixture of diastereoisomers, i.e. D-Man-(+)-oct. and D-Man-(-)-oct. (whose retention times are the same as the corresponding enantiomers, L-Man-(-)-oct. and L-Man-(+)-oct.), and D-Man-(-)-oct., respectively. The comparison of the three chromatograms allowed the identification of the D-configuration of both mannoses present on *C. pacifica* lipid A

In order to acquire structural information on the lipid A portion, an aliquot of purified LPS was subjected to a mild acid hydrolysis followed by centrifugation to isolate the lipid A as the water-insoluble precipitate. After further steps of purification, an aliquot of the lipid A was analyzed via MALDI-TOF MS and MS/MS, while another aliquot underwent a methanolysis step followed by acetylation to analyze fatty acids as methyl ester derivatives and to define the lipid A sugar backbone by inspecting the corresponding acetylated methyl glycosides. This analysis confirmed the fatty acid content obtained on full LPS (Table 1) as well as the presence of 2-amino-2-deoxy-glucose (glucosamine) and mannose (Fig. 1a) on the lipid A sugar backbone, as previously observed for other *Cellulophaga* strains [28]. Also in this case, absolute configuration of mannose and glucosamine was assigned as D (Fig. 1b).

### MALDI-TOF MS and MS/MS of lipid A from *C. pacifica* KMM 3664^T^

**Fig. 2:**
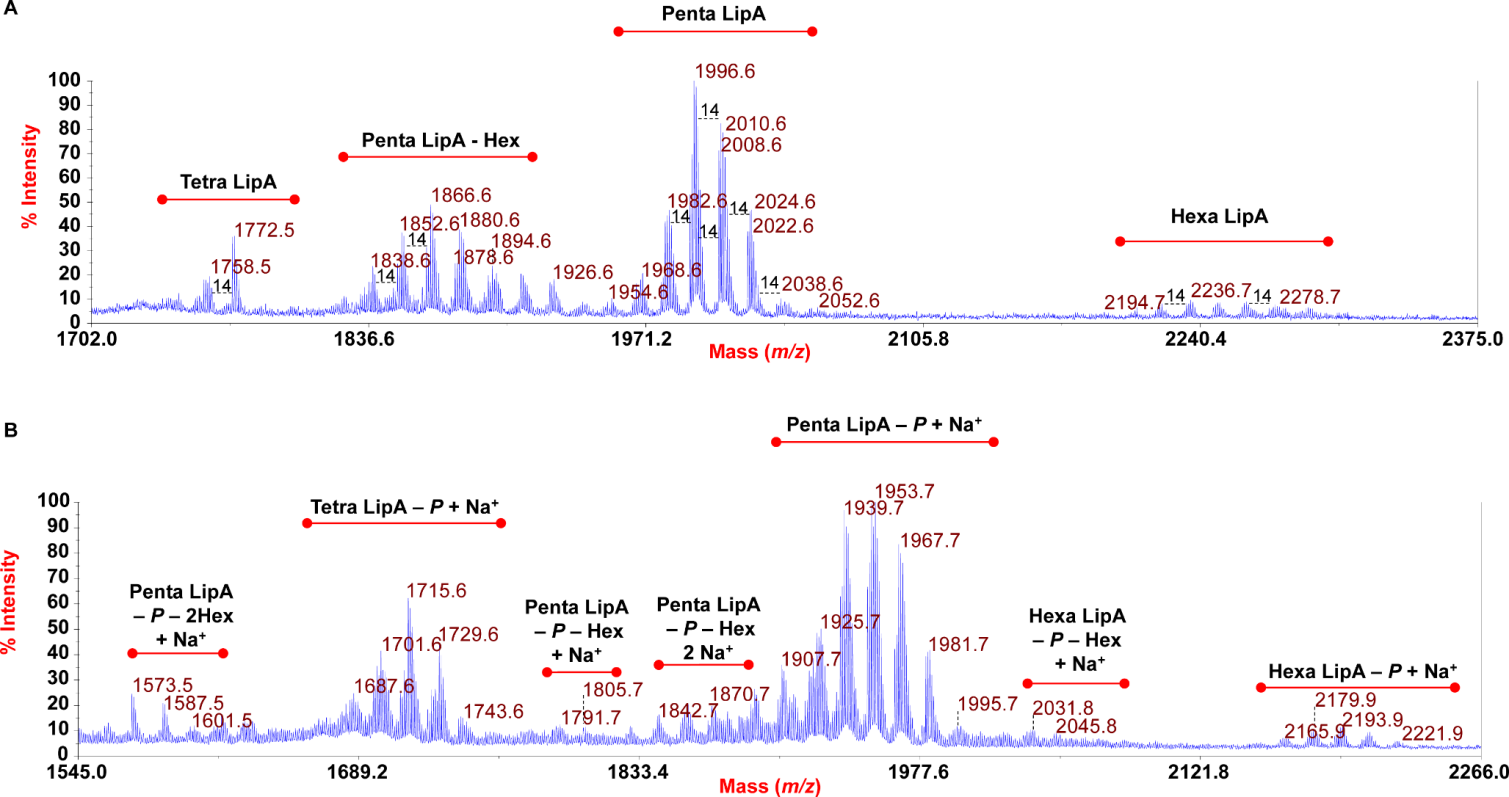
Reflectron MALDI-TOF mass spectra, recorded in negative (**a**) and positive (**b**) polarity, of lipid A from *C. pacifica* KMM 3664^T^ obtained after mild acid hydrolysis of purified LPS. The lipid A species are labelled as Tetra-, Penta- and Hexa Lip A indicating the degree of acylation. “Hex” stands for hexose, whereas “*P*” indicates the phosphate group

Reflectron MALDI-TOF mass spectra, recorded in negative and positive ion polarity, of lipid A from *C*. *pacifica* KMM 3664^T^ are shown in Fig. 2. Negative ion MALDI-TOF MS spectrum (Fig. 2a; Table 2) displayed the occurrence of two main clusters of peaks that were assigned to [M-H]^−^*mono*-phosphorylated tetra-acylated (at *m/z* 1772.5) and penta-acylated lipid A species (at *m/z* 1996.6) all carrying on the typical diglucosamine backbone a mannose disaccharide. A minor group of peaks was also detected at *m/z* 2236.7 and matched with *mono*-phosphorylated hexa-acylated lipid A species decorated by a dimannose unit (Fig. 2a; Table 2). Notably, each of these families of lipid A species displayed an additional level of complexity and heterogeneity due to the presence of peaks differing in 14 amu (a -CH_2_- unit) and 2 amu, which were diagnostic for the presence of lipid A forms that differ in the length of their acyl chain moieties and the occurrence of unsaturated fatty acids, respectively. Increasing the complexity of this lipid A, an intense cluster of peaks at *m/z* 1866.6 was also identified and assigned to *mono*-phosphorylated penta-acylated lipid A species.

**Table 2:** The main ion peaks observed in the MALDI-TOF MS spectra reported in Fig. 2, the predicted masses, and the proposed interpretation of the substituting fatty acids, phosphate (*P*) and mannose (Man), on the lipid A glucosamine backbone. The observed masses are compared to the calculated monoisotopic mass (predicted mass, Da) of each ion based on the proposed lipid A structures

**Predicted** **mass (Da)**	**Observed** **ion peaks (** ***m/z*** **)**	**Acyl substitution**	**Proposed composition**
***Negative-ion polarity***			
2236.6	2236.7	Hexa-acyl	HexN^2^, *P*, Man^2^, [17:0(3-OH)]^2^, [15:0(3-OH)]^2^, 17:0, 15:0
1996.4	1996.6	Penta-acyl	HexN^2^, *P*, Man^2^, [17:0(3-OH)]^2^, [15:0(3-OH)], 17:0, 15:0
2010.4	2010.6	Penta-acyl	HexN^2^, *P*, Man^2^, [17:0(3-OH)]^2^, [15:0(3-OH)], 17:0, 16:0
1982.4	1982.6	Penta-acyl	HexN^2^, *P*, Man^2^, [17:0(3-OH)]^2^, [15:0(3-OH)], 16:0, 15:0
1880.3	1880.6	Penta-acyl	HexN^2^, *P*, Man, [17:0(3-OH)]^3^, [16:0(3-OH)], [15:0(2-OH)]
1866.3	1866.6	Penta-acyl	HexN^2^, *P*, Man, [17:0(3-OH)]^2^, [16:0(3-OH)]^2^, [15:0(2-OH)]
1772.2	1772.5	Tetra-acyl	HexN^2^, *P*, Man^2^, [17:0(3-OH)]^2^, [15:0(3-OH)], 17:0
1758.2	1758.5	Tetra-acyl	HexN^2^, *P*, Man^2^, [17:0(3-OH)]^2^, [15:0(3-OH)], 16:0
***Positive-ion polarity***			
2194.6	2193.9	Hexa-acyl	HexN^2^, Man^2^, [17:0(3-OH)]^2^, [15:0(3-OH)]^2^, 17:0, 16:0, Na^+^
2046.6	2045.8	Hexa-acyl	HexN^2^, Man, [17:0(3-OH)]^2^, [15:0(3-OH)]^2^, (17:0)^2^, Na^+^ or HexN^2^, Man, [17:0(3-OH)]^2^, [15:0(3-OH)], [16:0(3-OH)], 17:0, 16:0, Na^+^
1954.4	1953.7	Penta-acyl	HexN^2^, Man^2^, [17:0(3-OH)]^2^, [15:0(3-OH)], 17:0, 16:0, Na^+^
1940.4	1939.7	Penta-acyl	HexN^2^, Man^2^, [17:0(3-OH)]^2^, [15:0(3-OH)], 17:0, 15:0, Na^+^
1842.3	1842.7	Penta-acyl	HexN^2^, Man, [17:0(3-OH)]^2^, [16:0(3-OH)], (17:0)^2^, 2Na^+^
1792.4	1791.7	Penta-acyl	HexN^2^, Man, [17:0(3-OH)]^2^, [15:0(3-OH)], 17:0, 16:0, Na^+^
1716.2	1715.6	Tetra-acyl	HexN^2^, Man^2^, [17:0(3-OH)]^2^, [15:0(3-OH)], 17:0, Na^+^
1730.2	1729.6	Tetra-acyl	HexN^2^, Man^2^, [17:0(3-OH)]^2^, [16:0(3-OH)], 17:0, Na^+^
1588.2	1587.5	Tetra-acyl	HexN^2^, [17:0(3-OH)]^2^, [15:0(3-OH)], (15:0)^2^, Na^+^

carrying only one D-mannose unit and a higher degree of hydroxylated fatty acids compared to the counterpart at *m/z* 1996.6. Briefly, the main peak at *m/z* 1996.6 was assigned to a penta-acylated lipid A species made up of the typical glucosamine disaccharide backbone substituted by one phosphate and a disaccharide of D-mannose, and carrying two 17:0(OH) [or *i*17:0(OH) or *a*17:0(OH)], one 15:0(OH) [or *i*15:0(OH) or *a*15:0(OH)], one 17:0 [or *i*17:0 or *a*17:0] and one 15:0 [or *i*15:0 or *a*15:0] (Table 1); related to this, the peak at *m/z* 1772.5 matched with the same lipid A species as above but devoid of the secondary 15:0 [or *i*15:0 or *a*15:0] unit. Hereafter the acyl moieties will be mentioned without specifying their linear or branched nature, taking into account that most of them can be also found in their *anteiso*- and *iso*-branched structures (Table 1). Accordingly, the positive-ion spectrum (Fig. 2b; Table 2) was remarkably complex and showed several clusters of peaks where the main one, at about *m/z* 1953.7, was assigned to [M-*P* + Na^+^]^+^ lipid A species, whose related cluster ascribable to tetra-acylated lipid A forms (i.e. devoid of one secondary fatty acid) was detected at about *m/z* 1715.6.

**Fig. 3:**
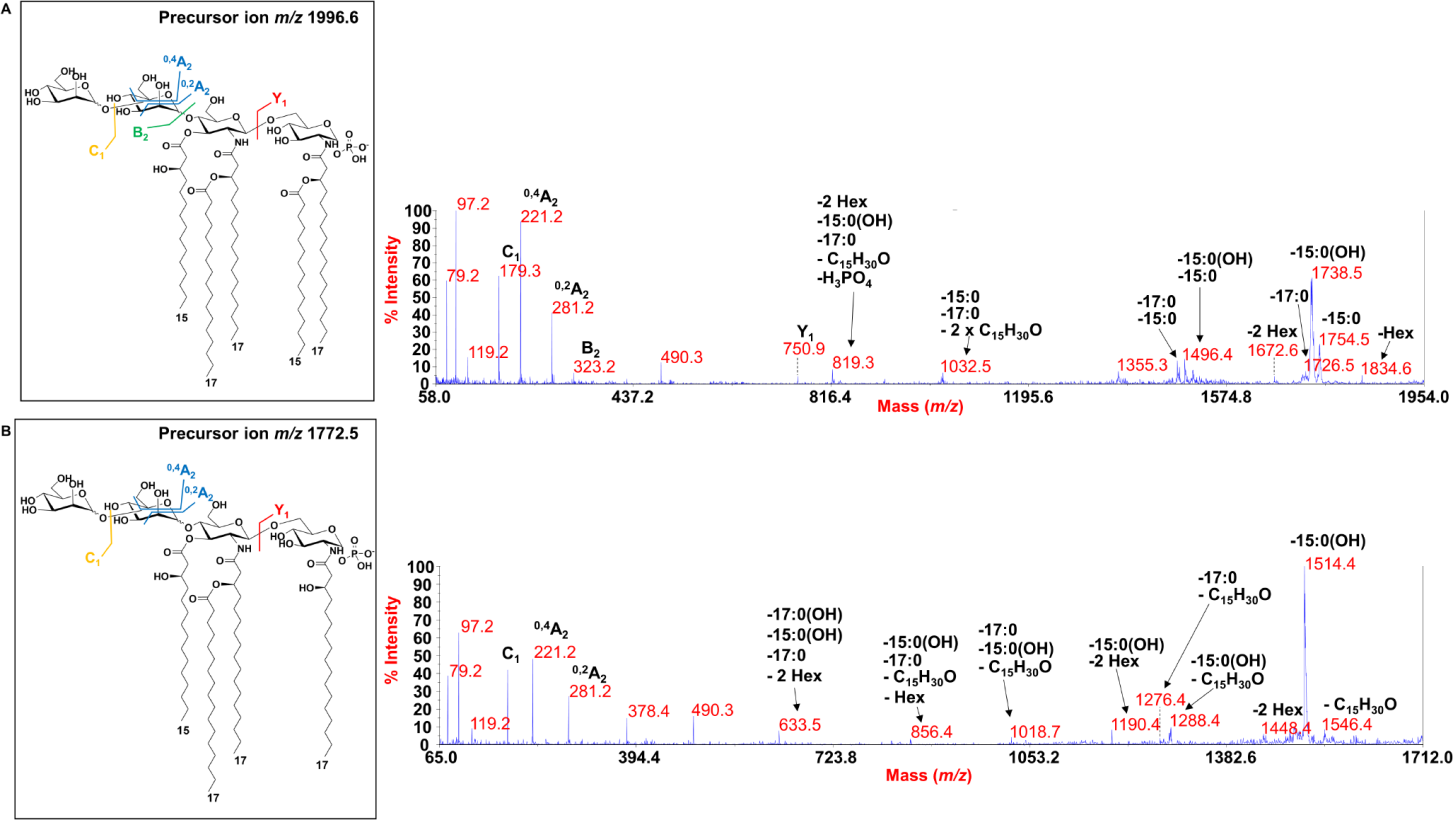
(**a**)Negative-ion MALDI-TOF MS/MS spectrum of precursor ion at *m/z* 1996.6, chosen as a representative ion peak of the cluster ascribed to penta-acylated lipid A species identified for *C. pacifica* KMM 3664^T^. (**b**)Negative-ion MALDI-TOF MS/MS spectrum of precursor ion at *m/z* 1772.5, a representative ion peak of the cluster assigned to tetra-acylated lipid A species. The assignment of main fragments is shown in both spectra. Peaks originated by the loss of C_15_H_30_O (226 mass units) following the rearrangement occurring on *N*-linked 3-OH acyl chains having the hydroxyl group free, have also been reported. The proposed structure of each lipid A is reported in the insets where the acyl chains were depicted as unbranched for description purposes only. The anomeric configuration of the D-mannose disaccharide as well as the linkage remain to be defined. Key fragmentations involving the mannose disaccharide are also reported in each inset

A negative-ion MS/MS investigation was carried out to disclose the exact location of the lipid A acyl chains with respect to the glucosamine disaccharide backbone as well as the position of the phosphate and the mannose residues. In detail, the MS/MS spectrum of the precursor ion at *m/z* 1996.6 (Fig. 3a) displayed two intense peaks at *m/z* 1738.5 and *m/z* 1754.5 that were assigned to ions originated from the loss of a 15:0(OH) and a 15:0, respectively, whereas a minor peak at *m/z* 1726.5 was attributed to the loss of a 17:0 unit. Less intense peaks were detected at *m/z* 1834.6 and *m/z* 1672.6 that matched with the loss of 162 amu and 324 amu respectively from the precursor ion, and therefore assigned to ions generated by the loss of a mannose (*m/z* 1834.6) and of the whole mannose disaccharide (*m/z* 1672.6). The observation of the ion peak at *m/z* 1496.4, designated as a fragment arisen from the sequential loss of 15:0(OH) and 15:0, was relevant for the structural deduction as it gave a first indication that the 15:0 unit was not bound as a secondary substituent of the primary ester linked 15:0(OH). On the other hand, the absence of any peak indicative of the loss of a whole unit of a hydroxylated fatty acid bearing 15:0 or 17:0 guided the placement of these moieties as secondary substituents of the primary *N*-linked fatty acids. In support to this hypothesis, it was crucial to also detect the ion at *m/z* 1032.6 originated from the sequential loss of 15:0 and 17:0 plus 452 amu. The loss of 452 amu was justified with a rearrangement that can occur only when the *N*-linked acyl chains do not bear secondary fatty acids, i.e. they have a free 3-OH group. In fact, an enamine to imine tautomerization followed by six-membered ring-based rearrangement can generate the loss of a C_15_H_30_O neutral fragment (226 amu) from each primary amide-linked 17:0(OH) acyl chain having a free 3-OH group [35, 36]. Since such a rearrangement was observed only when 15:0 and 17:0 were absent, the occurrence of the peak at *m/z* 1032.6 unequivocally located 15:0 and 17:0 as secondary acyl substituents of the 17:0(3-OH) moieties. Finally, the presence of the Y-type ion [37] at *m/z* 750.9, originating from the cleavage of the glycosidic bond of the two glucosamines, was fundamental to place the 15:0 unit on the reducing glucosamine together with one 17:0(OH) and the phosphate group; which concurrently located the 17:0 on the nonreducing glucosamine. In addition, the MS/MS spectrum also provided information about the dimannose unit which was placed on the nonreducing glucosamine. Indeed, some diagnostic peaks related to the mannose disaccharide were identified at *m/z* 179.3, 221.2 and 281.2, where the ion at *m/z* 179.3 was attributed to the external non-reducing mannose containing the glycosidic oxygen (C_1_ ion), whereas the ions at *m/z* 221.2 and *m/z* 281.2 were originated by the cross-ring fragmentations ^0,4^A_2_ and ^0,2^A_2_ respectively, occurring on the internal mannose unit. Similarly, the structure of the lipid A species detected at *m/z* 1772.5 (Fig. 3b) was determined as a tetra-acylated lipid A carrying two primary amide-linked 17:0(3-OH), one primary ester-bound 15:0(3-OH) and one 17:0 in a 3 + 1 symmetry with the respect to the glucosamine backbone that still carries a dimannose unit on the nonreducing glucosamine and a phosphate on the reducing end.

**Fig. 4:**
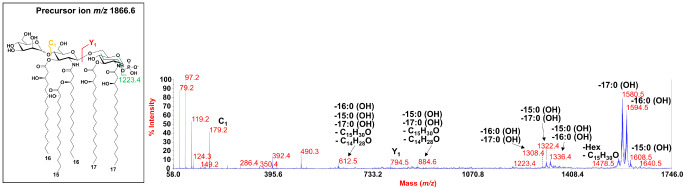
Negative-ion MALDI MS/MS spectrum of precursor ion at *m/z*1866.6, another representative ion peak of the cluster that matched with penta-acylated lipid A species but carrying only one D-mannose. The assignment of main fragments is reported. In the inset it is reported the proposed lipid A structure where the acyl chains were depicted as unbranched for description purposes only. The anomeric configuration of the D-mannose disaccharide as well as the linkage remain to be defined. Peaks arisen from the loss of C_15_H_30_O (226 mass units) have also been indicated as well as the fragmentations involving the mannose unit

To gain structural insights into the cluster of peaks ascribed to *mono*-phosphorylated penta-acylated lipid A species carrying only one D-mannose unit, the precursor ion at *m/z* 1866.6 (Fig. 2a) was chosen as representative for negative-ion MS/MS investigation. Briefly, the MS/MS spectrum (Fig. 4) showed intense peaks at *m/z* 1580.5 and *m/z* 1594.5, and a less intense peak at *m/z* 1608.5 which matched with ions derived by the loss of one 17:0(OH), one 16:0(OH), and one 15:0(OH), respectively. A family of peaks ascribable to ions originated by the sequential loss of two of the above hydroxylated fatty acids were also identified at *m/z* 1308.4, *m/z* 1322.4, and *m/z* 1336.4. The observation of the Y-type ion [37] at *m/z* 794.5 indicated the occurrence of the phosphate and two 17:0(OH) moieties on the reducing glucosamine which was further supported by the detection of the peak at *m/z* 1223.4 that arose from a cross-ring fragmentation involving the reducing glucosamine itself. Equally important for the structural characterization was the observation of the peaks at *m/z* 1640.5 and *m/z* 1478.5, which originated from the loss of 226 amu, due to the enamine to imine tautomerization occurring on the free primary *N*-linked 17:0(3-OH) (*m/z* 1640.5), and the loss of the hexose plus 226 amu (*m/z* 1478.5). Indeed, the occurrence of these peaks along with the Y-type ion at *m/z* 794.5 enabled the placement of the two 17:0(3-OH) as primary acyl chains of the reducing glucosamine. This structural deduction implied the occurrence on the nonreducing unit of two 16:0(OH), one 15:0(OH), and the mannose residue. Regarding the three hydroxylated fatty acids, these were necessarily located as primary acyl moieties for the two 16:0(OH), while the 15:0(OH) was placed as a secondary substituent in an acyloxyacyl amide moiety. Three main observations were crucial for this structural conclusion: (i) the absence of any peak indicative of the loss of a whole unit comprising two hydroxylated fatty acids; (ii) the observation of the peak at *m/z* 1608.5 (loss of 15:0(OH)), which was less intense than the one at *m/z* 1594.5 (loss of 16:0(OH)), which is justified by the weaker tendency of secondary acyl substituents of amide-linked fatty acids to be lost in MS/MS investigation; finally, (iii) fatty acid compositional analysis revealed the occurrence of 15:0(2-OH), which are typically carried as secondary substituents [38–41], as also observed in our previous studies on other *Cellulophaga* strains [28]. In conclusion, the lack of primary *O*-linked fatty acids on the reducing glucosamine in the penta-acylated lipid A species guided the placement of the sixth acyl chain of the very minor hexa-acetylated lipid A forms (cluster at about *m/z* 2236.7, Figure 2a) in such a position. However, its location remains to be confirmed.

Therefore, the combination of data from chemical analyses, MALDI-TOF MS and MS/MS investigation of lipid A enabled the definition of the structure of *C*. *pacifica* KMM 3664^T^ lipid A, which resulted in a highly complex blend of D-mannose disaccharide containing *mono*-phosphorylated tetra- to hexa-acylated lipid A species variously acylated by saturated, unsaturated, branched and unbranched fatty acids and also experiencing secondary 2-hydroxylation.

### TLR activation by LPS from *C. pacifica* KMM 3664^T^

**Fig. 5:**
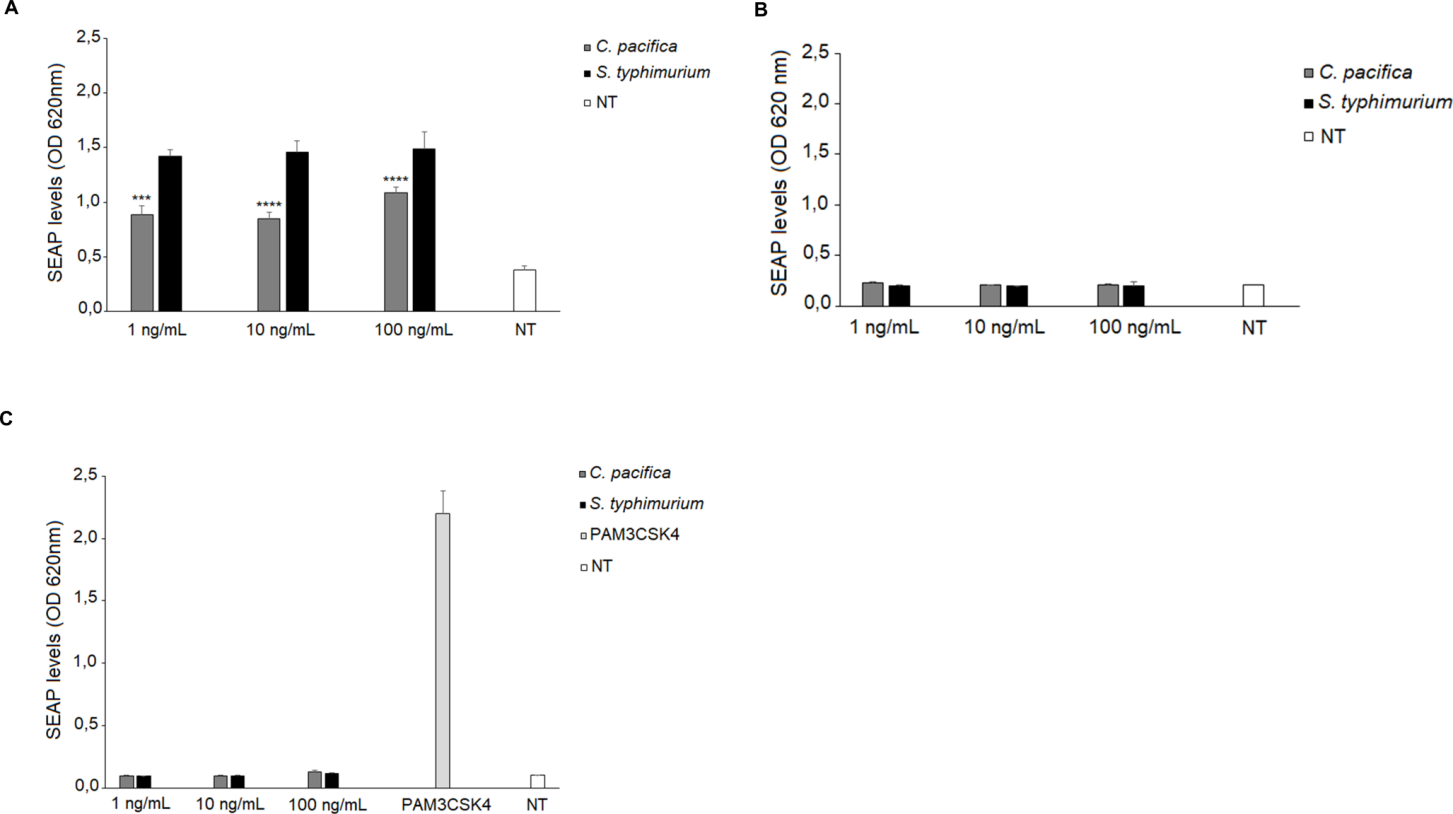
Stimulation of HEK Blue™ hTLR4 (**a**), HEK Blue™ Null2™ cells (**b**), and HEK Blue™ hTLR2 (**c**) cells. SEAP levels (OD) upon stimulation with LPS of *C. pacifica* KMM 3664^T^ at 1, 10, and 100 ng/mL; *S. typhimurium* SH 2201 S-LPS was used as the positive control. Significant differences between *C. pacifica* LPS and *S. typhimurium* S-LPS values are indicated. *** *p* < 0.001 and **** *p* < 0.0001 by the Student *t*-test. NT, not treated cells. Data are expressed as mean ± SD of three independent experiments in triplicate

The astounding heterogeneity of *C. pacifica* KMM 3664^T^ lipid A boosted our interest in investigating the immunostimulatory potential of the whole LPS by analyzing its effects on the activation of the immune receptor devoted to the recognition of the lipid A moiety, i.e. the TLR4/MD-2. To this aim, we used HEK-Blue™ cells expressing human TLR4 and MD-2/CD14, which enables to evaluate the activation of the NF-kB signal transduction pathway in turn responsible for regulating the expression of various pro-inflammatory genes and therefore representing a key mediator of the inflammatory response. HEK-Blue™ hTLR4 cells were treated with different concentrations of *C. pacifica* KMM 3664^T^ LPS (1, 10 and 100 ng/mL) while *S. typhimurium* SH 2201 LPS, at the same concentrations, was used as the positive control. Untreated cells were instead the negative control for these experiments. The significantly weaker NF-kB activation upon stimulation with *C*. *pacifica* KMM 3664^T^ LPS compared to *S. typhimurium* LPS was immediately apparent at all concentrations tested (*C. pacifica* vs. *S. typhimurium* SH 2201 LPS, *p*-value < 0.001 at 1 ng/mL and *p*-value < 0.0001 at 10 and 100 ng/mL, Fig. 5a). To further validate these results, we performed the same experiments in HEK-Blue™Null2™ cells, i.e. the parental cell lines of HEK-Blue™ hTLR4 but lacking in TLR4/MD-2/CD14 expression (Fig. 5b). No NF-kB activation was detected in these cells treated with *C. pacifica* KMM 3664^T^ LPS, thus demonstrating that this LPS induced NF-kB activation via TLR-dependent mechanism.

Furthermore, to evaluate any potential involvement of other TLRs, besides excluding the presence of any possible contamination by other immunostimulant molecules (such as lipoproteins), we also stimulated HEK-Blue™ hTLR2 cells with *C. pacifica* KMM 3664^T^ or *S. typhimurium* LPS. In this experiment, the treatment with Pam3CSK4 (PAM), a TLR2 agonist, was used as the positive control. Also in this case, no statistical differences were observed in the NF-kB activation upon stimulation with *C. pacifica* KMM 3664^T^ LPS respect to the negative control, thus proving that this LPS did not activate TLR2-mediated signaling and confirming the absence of any other immunostimulatory molecule co-extracted with the LPS (Fig. 5c).

## Discussion

Stressful conditions of salinity, temperature variation, desiccation and light exposure influence the chemical structure of the membrane constituents of Gram-negative bacteria thriving marine waters [42]. Under these selective pressures, the membrane anchor of marine bacterial LPS, the lipid A, can dramatically change and exhibit chemical features remarkably different from those found in their terrestrial counterparts. Here we show that, like other *Cellulophaga* strains, *C. pacifica* KMM 3664^T^ produces a highly diverse blend of lipid A species spanning from tetra- to hexa-acylated and all carrying one phosphate and one or two D-mannose residues on the glucosamine backbone. Briefly, the main species detected was a penta-acylated lipid A carrying a disaccharide of D-mannose on the nonreducing glucosamine and one phosphate on the reducing unit while bearing two primary *N*-linked 17:0(OH), one primary *O*-linked 15:0(OH), and 15:0 and 17:0 as secondary acyl substituents. Nevertheless, an astounding heterogeneity in the nature (length and saturation degree) of the acyl chains generated a huge number of co-existing diverse *mono*-phosphorylated and dimannose-containing penta-acylated lipid A molecules. Further enhancing such a complex structural heterogeneity, *C. pacifica* KMM 3664^T^ also showed to synthesize highly diverse and 2-hydroxylation experiencing lipid A species carrying only one D-mannose unit, as previously observed in other *Cellulophaga* strains [28]. Therefore, several chemical features seem to be peculiar of this genus: the high degree of branched and unsaturated acyl chains, the occurrence of fatty acids containing 15 and 17 (i.e. odd numbered) carbon atoms, and the presence of D-mannose in place of one phosphate group. The occurrence of all these structural peculiarities might be attributed to adaptive evolution mechanisms developed to maintain the integrity of bacterial cell membrane when challenged to grow in highly mutable environmental conditions. Indeed, it is largely known that lipid A fatty acids are important modulators of Gram-negative outer membrane properties [43, 44]. Membrane fluidity in fact can be adjusted by altering the degree of lipid packing, which in turn affects the water permeability across the membrane [45]. In this frame, saturated acyl chains are responsible for a tighter and denser packing compared to unsaturated chains, thus resulting in a more rigid membrane structure. By contrast, the presence of monounsaturation, which gives rise to a slight curled configuration, results in a “relaxation” of the membrane thus increasing membrane fluidity. Likewise, the presence of branched fatty acids (i.e. of methyl groups) as well as an increase in the hydroxylation pattern also impacts on the fluidity of the membrane by affecting the melting temperature [45, 46]. As a matter of fact, *C. pacifica* KMM 3664^T^ seems to adopt all the above strategies for survival and persistence in the marine environment by locating these chemical modifications in its lipid A moiety. Strikingly, *C. pacifica* KMM 3664^T^ provides an additional level of complexity that is represented by the substitution of one phosphate group with a disaccharide of D-mannose, which was in contrast to other *Cellulophaga* lipid As that only carry one D-mannose unit [28]. In general, this modification might be associated with an enhancement of membrane stability as a glycosidic linkage is stronger than a phosphate ester bond, thus likely assuring survival in stress conditions. That said, the definition of the molecular mechanisms at the basis of these membrane chemical modifications is pivotal to have a comprehensive picture of the microbial adaptation phenomenon. Shedding light on these mechanisms will also explain the significant differences in fatty acid nature/distribution and in phosphate presence/substitution that can be observed even between species of the same marine bacterial genus.

**Fig. 6:**
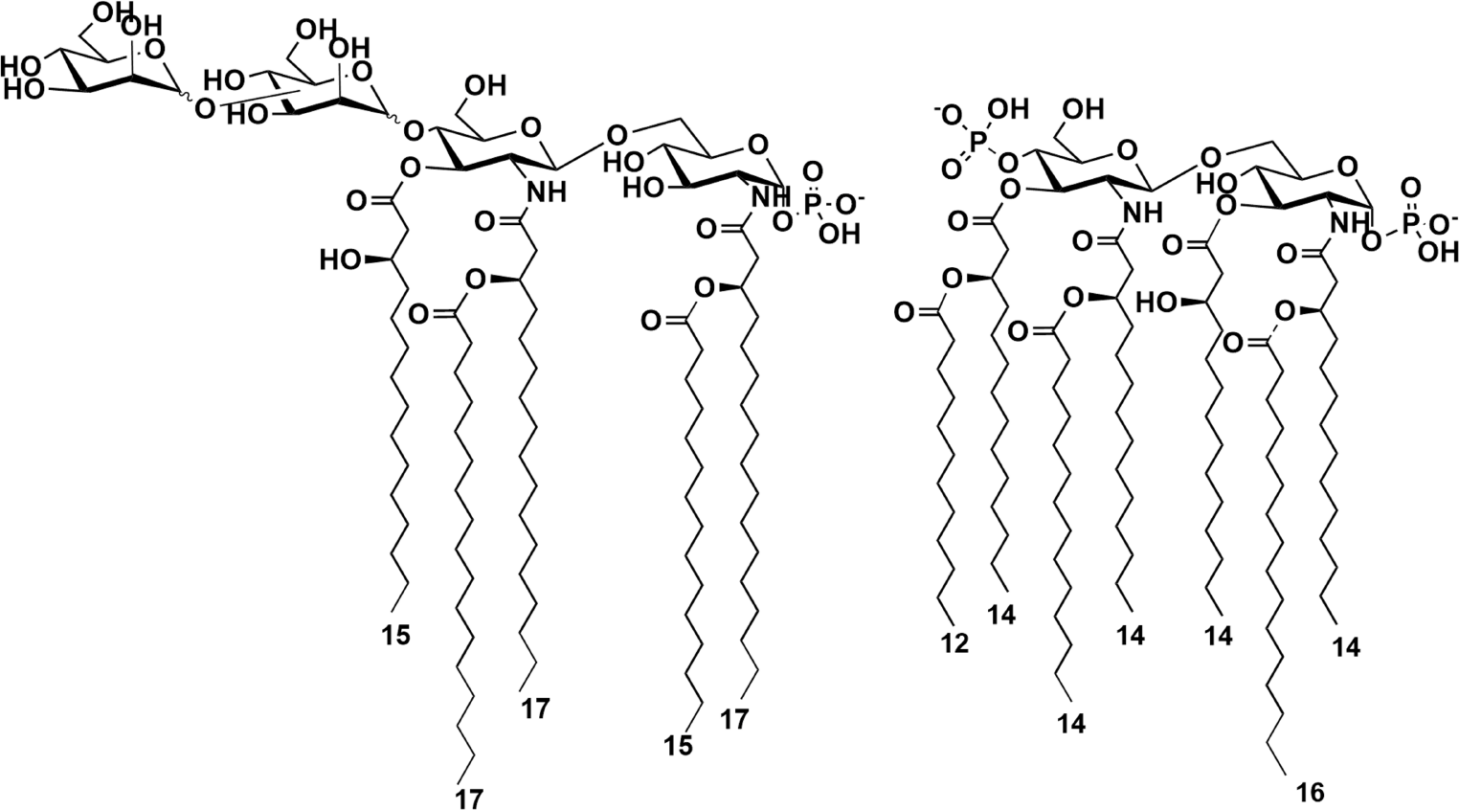
Structural comparison of main lipid A species of *C. pacifica* LPS (*m/z* 1996.6) (*left*) and *S. typhimurium* SH 2201 LPS (*right*), which was used as the positive control in biological assays. Dotted lines indicate a non-stoichiometric substitution

Intrigued by the unusual structural features and the astounding structural heterogeneity of *C. pacifica* KMM 3664^T^ lipid A, we have evaluated the potential of the whole LPS in activating TLR4-dependent signaling by leveraging HEK-Blue cells expressing human TLR4, MD-2, and CD-14. We have demonstrated that *C. pacifica* KMM 3664^T^ LPS acts as a weaker stimulator of the TLR4-mediated immune response compared to the enteropathogenic *S. typhimurium* LPS. This behavior was rather expected as the result of the hypo-acylation and hypo-phosphorylation of the main lipid A species composing *C. pacifica* KMM 3664^T^ LPS compared to *S. typhimurium* LPS whose lipid A is *bis*-phosphorylated and carries six or seven acyl chains (Fig. 6). Indeed, it is largely acknowledged that lipid A expressing less than six acyl chains usually only poorly activate the TLR4-mediated signaling. Yet, we have highlighted that *C. pacifica* KMM 3664^T^ also synthesizes hexa-acylated lipid A species, which however were less abundant and in a 3 + 3 distribution with the respect to the glucosamine backbone, which in turn is also commonly associated with a lower immunogenicity compared to the 4 + 2 symmetry [14]. Likewise, the low degree of phosphorylation observed for *C. pacifica* KMM 3664^T^ lipid A might further contribute to the decreased TLR4 activation, as also previously observed [47].

## Conclusions

In this study, we have determined the structure of the lipid A from *C. pacifica* KMM 3664^T^, a Gram-negative marine bacterium belonging to the family Flavobacteriaceae of the phylum Bacteroidetes that was isolated from the Sea of Japan. We have shown that this bacterium can synthesize a highly diverse and complex family of lipid A species, all *mono*-phosphorylated and mainly carrying five acyl chains. Hexa-acylated lipid A species with a symmetric distribution of the fatty acids were also identified as minor components, like tetra-acylated forms. Notably, most of the lipid A species also carry a disaccharide of D-mannose on the glucosamine backbone in place of the typical phosphate group, as previously observed for plant associated and environmental bacteria [35, 36, 48–50], thus decreasing the overall negative charge of the lipid A itself. Increasing the complexity of the *C. pacifica* KMM 3664^T^ outer membrane, most of these lipid As also experience unsaturation, high degree of branching as well as 2-hydroxylation of the acyl chains. Overall, this study further proved the extraordinary heterogeneity of lipid A molecules from marine bacteria, with chemical differences also appreciable among species within the same genus. Moreover, given the weak immunostimulatory capacity of *C. pacifica* KMM 3664^T^ LPS, with this study we hope to encourage the research and analysis of LPSs from aquatic environment that can be considered an almost infinite source of natural substances with immunomodulatory properties and with several potential biomedical applications such as immune therapeutics and vaccine adjuvants [51].

## Data Availability

No datasets were generated or analysed during the current study.

## Abbreviations

AMG: acetylated methyl glycoside
DMEM: Dulbecco’s minimal essential media
FBS: fetal bovine serum
GC-MS: Gas Chromatography-Mass Spectrometry
GlcN: 2-amino-2-deoxy-D-glucose
LPS: Lipopolysaccharide
MALDI: Matrix-Assisted Laser Desorption Ionization
MD-2: Myeloid Differentiation factor-2
MS: Mass Spectrometry
NF-κB: nuclear factor kappa-light-chain-enhancer of activated B cells
PAM: Pam3CSK4
R-LPS: Rough-type Lipopolysaccharide
SEAP: secreted alkaline phosphatase
SDS-PAGE: Sodium Dodecyl Sulfate-Polyacrylamide Gel Electrophoresis
S-LPS: Smooth-type Lipopolysaccharide
THAP: 2′,4′,6′-Trihydroxyacetophenone
TLR4: Toll-Like Receptor 4
TLR2: Toll-Like Receptor 2
TOF: Time Of Flight
